# Benefits and harms of cervical screening, triage and treatment strategies in women living with HIV

**DOI:** 10.1038/s41591-023-02601-3

**Published:** 2023-12-12

**Authors:** Michaela T. Hall, Kate T. Simms, John M. Murray, Adam Keane, Diep T. N. Nguyen, Michael Caruana, Gigi Lui, Helen Kelly, Linda O. Eckert, Nancy Santesso, Silvia de Sanjose, Edwin E. Swai, Ajay Rangaraj, Morkor Newman Owiredu, Cindy Gauvreau, Owen Demke, Partha Basu, Marc Arbyn, Shona Dalal, Nathalie Broutet, Karen Canfell

**Affiliations:** 1 Daffodil Centre, University of Sydney, a joint venture with Cancer Council NSW, Sydney, New South Wales Australia; 2 School of Mathematics and Statistics, University of New South Wales, Sydney, NSW Australia; 3 London School of Hygiene and Tropical Medicine, University of London, London, UK; 4 Department of Global Health and the Department of Obstetrics & Gynecology, University of Washington, Seattle, WA USA; 5 Department of Health Research Methods, Evidence and Impact, McMaster University, Hamilton, Ontario Canada; 6 Division of Cancer Epidemiology and Genetics, National Cancer Institute, Rockville, MD USA; 7 ISGlobal, Barcelona, Spain; 8 Universal Health Coverage and Life Course Cluster, World Health Organization, Dar es Salaam, Tanzania; 9 Department of Global HIV, Hepatitis and Sexually Transmitted Infections Programmes, World Health Organization, Geneva, Switzerland; 10 Child Health Evaluative Sciences, The Hospital for Sick Children Research Institute, Toronto, Ontario Canada; 11 SUCCESS Project, Expertise France, Paris, France; 12 Global Diagnostics, Clinton Health Access Initiative, Kigali, Rwanda; 13 Early Detection Prevention and Infections, International Agency for Research on Cancer, Lyon, France; 14 Cancer Epidemiology Unit, Belgian Cancer Centre, Sciensano, Brussels, Belgium; 15 Department of Human Structure and Repair, Faculty of Medicine and Health Sciences, University of Ghent, Ghent, Belgium; 16 Department of Reproductive Health and Research, World Health Organization, Geneva, Switzerland

**Keywords:** Population screening, Cancer epidemiology, Cancer prevention, Cervical cancer

## Abstract

To support a strategy to eliminate cervical cancer as a public health problem, the World Health Organisation (WHO) reviewed its guidelines for screening and treatment of cervical pre-cancerous lesions in 2021. Women living with HIV have 6-times the risk of cervical cancer compared to women in the general population, and we harnessed a model platform (‘Policy1-Cervix-HIV’) to evaluate the benefits and harms of a range of screening strategies for women living with HIV in Tanzania, a country with endemic HIV. Assuming 70% coverage, we found that 3-yearly primary HPV screening without triage would reduce age-standardised cervical cancer mortality rates by 72%, with a number needed to treat (NNT) of 38.7, to prevent a cervical cancer death. Triaging HPV positive women before treatment resulted in minimal loss of effectiveness and had more favorable NNTs (19.7–33.0). Screening using visual inspection with acetic acid (VIA) or cytology was less effective than primary HPV and, in the case of VIA, generated a far higher NNT of 107.5. These findings support the WHO 2021 recommendation that women living with HIV are screened with primary HPV testing in a screen-triage-and-treat approach starting at 25 years, with regular screening every 3–5 years.

## Abstract (web summary)

A model evaluating cervical screening, triage and treatment strategies to prevent cervical cancer in women living with HIV in Tanzania provides evidence to support the World Health Organization’s recommendation of primary HPV testing starting at age 25 years with 3–5-year regular screening intervals.

## Main

The age-standardized rate of cervical cancer mortality among women living in low- and lower-middle-income countries (LMICs) is estimated to be 12.9–14.1 deaths per 100,000 women in 2020, with women in Eastern African regions subject to cervical cancer mortality rates of 28.6 deaths per 100,000 women^1,2^. Women living with HIV are disproportionately affected by cervical cancer; they have a sixfold increase in lifetime risk compared to other women and account for 5% of all cervical cancer cases, despite the global prevalence of HIV being less than 1%^3,4^.

The World Health Organization (WHO) has called all nations to implement a triple-intervention strategy that aims to eliminate cervical cancer as a public health problem^5^. This strategy recommends that countries implement the ‘90–70–90’ intervention targets by 2030, which are (1) 90% of girls fully vaccinated with the HPV vaccine by 15 years of age; (2) 70% of women screened using a high-performance test by 35 years of age and again by 45 years of age; and (3) 90% of women identified with cervical pre-cancer or invasive cervical cancer have access to adequate treatment and care^5^. Countries will subsequently be considered to have eliminated cervical cancer as a public health problem when rates of new cases fall below 4 per 100,000 women-years. Comprehensive modeling undertaken by the WHO Cervical Cancer Elimination Modelling Consortium (CCEMC) in 78 LMICs, which accounted for overall burden of disease but did not explicitly account for endemic HIV and HIV control, found that, if the 2030 triple-intervention targets are achieved in 78 LMICs, cervical cancer would be eliminated and a total of 74.1 million cancer cases and 62.6 million deaths would be averted over the course of the century^5^. Additional CCEMC analyses that explicitly included HIV and HPV interactions found that, although reaching the elimination threshold was possible in South Africa (another high HIV burden country), it was not possible among women living with HIV given only two lifetime screens in this group, although cervical cancer incidence approached the elimination threshold of 4 cases per 100,000 women^6,7^.

Recommendations for screening generally differ from those for the general population in women living with HIV, not only because of the higher disease burden but also because of the need to consider differences in screening test performance and pre-cancer treatment efficacy in this group, which may also vary by HIV disease stage and HIV viral suppression^4,8^. In 2013, the WHO provided guidance on Comprehensive Cervical Cancer Control for women with or without HIV^9^. In these previous guidelines, for women living with HIV or unknown HIV status in areas with high endemic HIV infection, the WHO recommended that screening intervals should be no longer than 3 years. However, to account for new advances in screening, triage and treatment technologies, in parallel with the launch of the elimination strategy in 2020, the WHO initiated the development of updated guidelines for screening and treatment for cervical cancer prevention^8,10,11^. Expert technical teams undertook a range of activities, including systematic reviews and modeling, with reference to an advisory group, the Guidelines Development Group for Screening and Treatment to Prevent Cervical Cancer. Clinical evidence was assessed separately for the general population of women and for women living with HIV^8^. A modeled evaluation of potential approaches to cervical screening was conducted separately for each group. This also drew upon an evidence review conducted for the 2021 International Agency for Research on Cancer Handbook of Cervical Screening^12^.

The aim of the modeled analysis reported here was to assess the benefits and harms of various screening approaches and to determine which combination of screening test technology, interval and age range is optimal in women living with HIV. This is complementary to a separately conducted modeled evaluation for the general population of women reported in a companion paper^8^.

## Results

We used the Policy1-Cervix-HIV platform, a deterministic transmission-dynamic compartment model of sexual behavior, HIV and HPV infection and natural history, which captures simultaneous HIV and multi-type HPV infections and incorporates comprehensive demographic, sexual behavior and natural history assumptions^13,14^. Outcomes over the lifetime (10–84 years) of Tanzanian females born in 2005 who acquire HIV on or before their 25th year of age were simulated to assess seven screening algorithms, including primary visual inspection with acetic acid (VIA), primary cytology and primary HPV with no triage or triage using HPV 16/18 genotyping, colposcopy, cytology or VIA. In the base case, we assumed 3-yearly screening intervals for primary VIA and cytology and intervals of 3 years, 5 years and 10 years for primary HPV (Table 1). Screening and triage test performance for the base case and the ranges considered for sensitivity analysis were informed by updated systematic review evidence. Furthermore, we assumed that 70% of women attended each routine screen and 90% attended follow-up or treatment. Outcomes included reduction in cancer incidence and mortality, number needed to treat (NNT) and pre-term delivery events directly due to pre-cancer treatment. Table 2 summarizes our main findings and the policy impact of this research.

**Table 1:** Screening scenarios considered in the evaluation

Screening and triage technology	Screening age, frequency and number of lifetime screening events
Primary VIA^a^	• 3 yearly, 25–50 years (9×)
Cytology, HPV triage for ASC-US^b^	
Primary HPV^a^	• 3 yearly, 25–50 years (9×) • 5 yearly, 25–50 years (6×) • 10 yearly, 25–50 years (3×) • 10 yearly, 30–50 years (3×) • 10 yearly, 35–45 years (2×) ‘WHO elimination strategy’^5^
HPV, HPV 16/18 triage^c^	
HPV, VIA triage^d^	
HPV, colposcopy triage	
HPV, cytology triage^b^	

^a^All HPV^+^ women treated after assessment of eligibility for ablative treatment for same-day ablation. ^b^HPV^+^ women or women with cytology > ASC-US referred to colposcopy. ^c^HPV 16/18-positive women treated after assessment of eligibility for ablative treatment and women positive for only other high-risk HPV types are treated only if VIA triage positive. ^d^VIA triage-positive women treated after assessment of eligibility for ablative treatment. The ‘WHO elimination strategy’ refers to the screening test, ages and frequencies assumed in the earlier analysis of the cervical cancer elimination timeline^2,6^.

**Table 2:** Policy summary

Background	Access to effective cervical cancer prevention in LMICs is currently limited, and women living with HIV are at a sixfold increased risk of cervical cancer. In 2020, the WHO launched a global strategy to eliminate cervical cancer as a public health problem and recommends ‘90–70–90’ intervention targets by 2030. These are that (1) 90% of girls are fully vaccinated against HPV by 15 years of age; (2) 70% of women are screened using a high-performance test by 35 years of age and again by 45 years of age; and (3) 90% of women identified with cervical pre-cancer or invasive cervical cancer are provided adequate treatment and care. To facilitate the implementation of the elimination strategy, the WHO updated its 2013 cervical screening and treatment guidelines in 2021 under the auspices of the Guidelines Development Group for Screening and Treatment to Prevent Cervical Cancer, which comprises a range of scientists, healthcare providers, implementers, ministry of health representatives, systematic reviewers, program implementation experts and representatives from civil society. A specific evidence review was performed for women living with HIV to inform the guidelines update for cervical screening in this population.
Main findings and limitations	In women living with HIV, primary HPV testing with triage at a 5-yearly interval was more effective at reducing cervical cancer cases and deaths than screening with VIA every 3 years. Screening with primary HPV testing every 3 years was the most effective option for reducing cervical cancer incidence. The inclusion of triaging strategies in HPV^+^ women living with HIV resulted in minimal loss in efficacy while simultaneously reducing the number of pre-cancer treatments by 11–52%, depending on the screening technology and interval. Therefore, the benefits of HPV screening can be realized while mitigating potential harms of overtreatment in this group by implementing HPV screening in a screen, triage and treat algorithm.
Policy implications	Based on evidence review, together with the findings of this analysis, the WHO has recommended using HPV as the primary screening test (rather than VIA or cytology) in women living with HIV. For women living with HIV, a 3–5-yearly screening interval offers an appropriate balance of benefits to harms. Although it is recommended that women in the general population receive HPV screening with or without triage, the WHO recommends implementing an appropriate triaging strategy for women living with HIV (HPV 16/18 genotyping, colposcopy, cytology or VIA) to reduce the expected overall burden and subsequent harm of overtreatment in this group. The development of practical and effective programmatic models of HPV screen, triage and treat for women living with HIV will depend on the availability of affordable HPV and triage tests, appropriate linkages with reproductive and HIV services and effective registry mechanisms for recalling women for surveillance follow-up or referring them for further management.

### Cervical cancer incidence and mortality

**Fig. 1:**
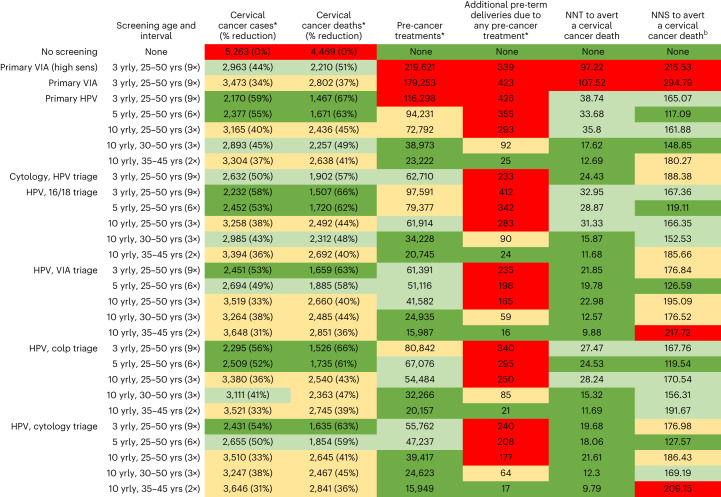
Summary of the number of cervical cancer cases, cervical cancer deaths, pre-cancer treatments, additional pre-term delivery events, NNTs and NNSs over the lifetime of a cohort of 100,000 women living with HIV. The cells are colored to provide an overall impression of strategies that are performing well: best-performing strategies in a column are colored green (best—largest cancer incidence/mortality reduction or the lowest number of pre-cancer treatments, NNTs or NNSs), followed by teal, yellow and then red for the worst-performing strategies. The range for the color coding for each column is shown in Extended Data Fig. 1. ^b^NNS to avert a cervical cancer death includes primary and follow-up testing. yrly, yearly; yrs, years; NNS, number needed to screen.

**Fig. 2:**
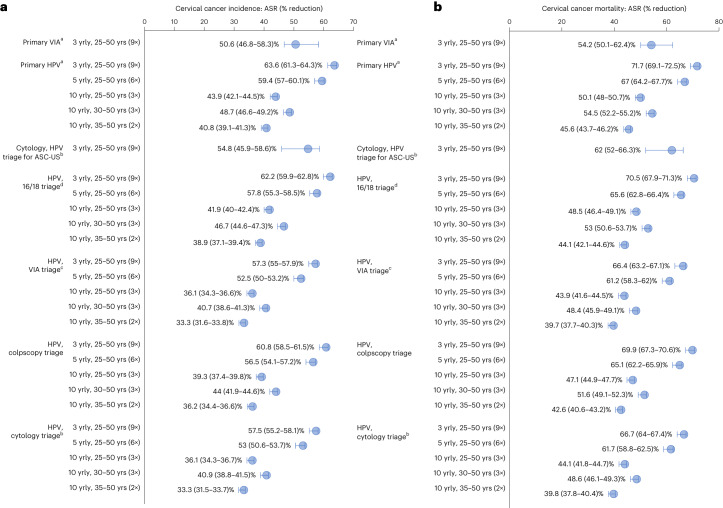
Effectiveness of simulated screening approaches. **a**,**b**, Reductions in ASIR (**a**) and ASMR (**b**) compared to no screening in women living with HIV, shown as the dots for baseline assumptions. The error bars around the dots represent the reductions when assuming the best (upper range) and worst (lower range) primary test performance assumptions as described in the Methods. Age standardization is calculated using the 2015 World Female Population for ages 0–99 years. ASR, age-standardized rate; yrly, yearly; yrs, years. ^a^All positive women treated after assessment of eligibility for ablative treatment for same-day ablation. ^b^ HPV positive women or women with cytology > ASC-US referred to colposcopy. ^c^VIA triage positive women treated after assessment of eligibility for ablative treatment. ^d^HPV 16/18 positive women treated after assessment of eligibility for ablative treatment and women positive for only other hrHPV types are treated only if VIA triage positive.

Without cervical screening, the predicted age-standardized incidence rates (ASIRs) and age-standardized mortality rates (ASMRs) for the simulated cohort were predicted to be 104 and 100, respectively, per 100,000. This corresponds to 5,263 cervical cancer cases and 4,469 deaths over the lifetime of this cohort (Fig. 1). In the base case, primary HPV testing without triage every 3 years for ages 25–50 years reduced age-standardized cervical cancer ASIR by 64% (Fig. 2); triaging HPV^+^ women before treatment reduced cervical cancer ASIR by 57–62% (range depends on triaging technology). Respectively, primary cytology with HPV triage and primary VIA testing, when offered every 3 years, could reduce cervical cancer ASIR by 55% and 51%.

Primary HPV screening every 5 years for ages 25–50 years without triage was predicted to reduce cervical cancer ASIR by 59%, which is 4.2 percentage points lower than screening every 3 years. Primary HPV testing every 5 years for ages 25–50 years with triage using 16/18 genotyping, cytology, VIA or colposcopy was predicted to result in a 4.4–4.8 percentage points lower reduction in cervical cancer ASIR compared to the equivalent 3-yearly strategy.

### Balance of benefits and harms

Primary HPV screening without triage every 3 years for ages 25–50 years was predicted to result in 116,298 pre-cancer treatments and 426 additional pre-term delivery events with an NNT of 38.7 (Fig. 1). Primary HPV screening with triaging with VIA, HPV 16/18 genotyping, cytology or colposcopy every 3 years was predicted to generate 55,762–97,591 pre-cancer treatments and 240–412 additional pre-term deliveries. Primary HPV testing every 3 years for ages 25–50 years with any of the triaging options also generated NNTs of 19.7–33.0. Primary VIA testing every 3 years generated 179,253–219,621 pre-cancer treatments over the lifetime of 100,000 women living with HIV and NNTs of 97–108.

Primary HPV screening every 5 years for ages 25–50 years, with or without triage, was predicted to result in 8,525–22,067 fewer pre-cancer treatments over the lifetime of the cohort compared to the equivalent strategy every 3 years (range dependent on triaging option). Screening every 5 years was predicted to result in 1.6–5.1 fewer NNTs compared to the equivalent strategy every 3 years.

**Fig. 3:**
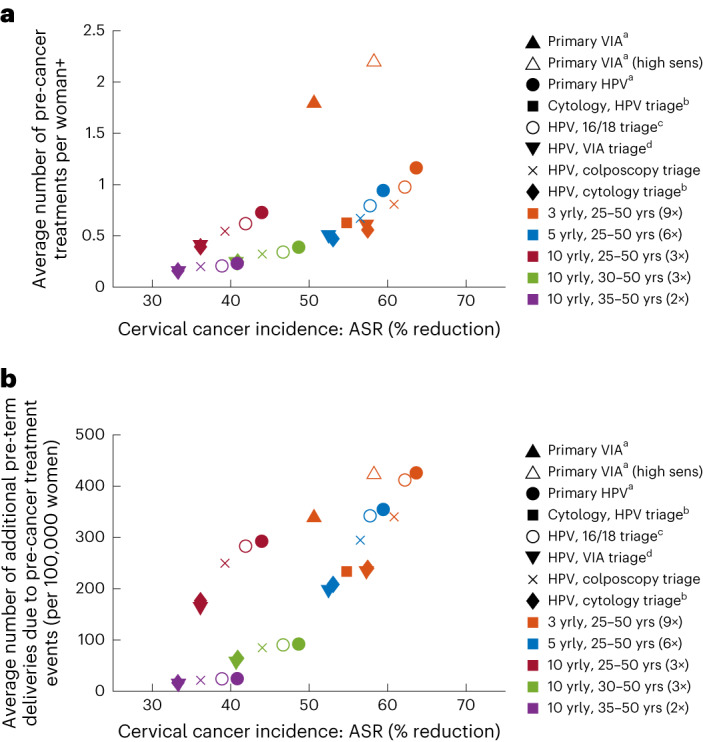
Benefits versus harms of simulated screening approaches. **a**,**b**, Cervical cancer incidence reduction versus average lifetime number of pre-cancer treatment events (**a**) and number of additional pre-term delivery events due to pre-cancer treatments per cohort of 100,000 women (**b**) for each screening approach in women living with HIV. ASR, age-standardized rate; yrly, yearly; yrs, years. ^a^All positive women treated after assessment of eligibility for ablative treatment for same-day ablation. ^b^HPV positive women or women with cytology > ASC-US referred to colposcopy. ^c^HPV 16/18 positive women treated after assessment of eligibility for ablative treatment and women positive for only other hrHPV are treated only if VIA triage positive +Note there could be multiple treatments in women who require follow-up. ^d^VIA triage positive women treated after assessment of eligibility for ablative treatment.

For women living with HIV, primary HPV screening without triage and primary HPV screening with HPV 16/18 genotyping triage, at a 3-yearly interval, resulted in slightly more additional pre-term deliveries than 3-yearly primary screening with VIA, despite primary HPV testing resulting in substantially fewer overall pre-cancer treatments per lifetime (Fig. 3). This result is mainly because the high sensitivity of an HPV test results in increased detection before age 30 years, overlapping with a period of high fertility (assumed median age at childbirth is 26 years), whereas a larger number of the additional treatments in primary VIA testing occur beyond age 30 years.

### Supplementary analysis

**Extended Data Fig. 2:**
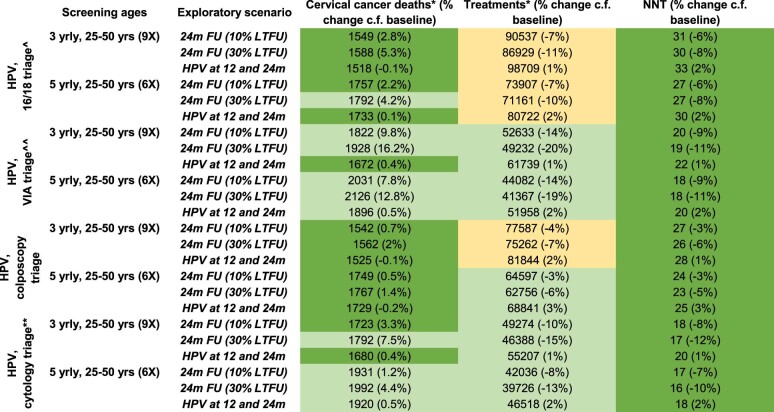
Summary of key outcomes for simulated screening approaches in supplementary analysis for triage management. Number of predicted cervical cancer deaths, precancer treatments and the number needed to treat to prevent a cervical cancer death per 100,000 Tanzanian women living with HIV, compared to baseline and no screening scenarios for supplementary analyses considering the management of HPV positive triage negative women. The range for the colour coding for each column is shown in Extended Data Fig. 1.

For screening approaches involving primary HPV screening, at screening intervals of 3-yearly or 5-yearly, referring women who test HPV postive and triage test negative to return in 24 months (30% loss to follow-up) results in 1–16% additional cervical cancer deaths and 7–20% fewer pre-cancer treatments over the lifetime of the cohort, relative to the base case (re-screening at 12 months, 10% loss to follow-up), depending on triage strategy. When referring women who test HPV positive and triage test negative to return in 24 months, but assuming that loss to follow-up was only 10%, we predict a 0.5–9.8% increase in cervical cancer deaths and 3–7% fewer pre-cancer treatments, relative to the base case (Extended Data Fig. 2).

In the supplementary scenario where women were followed-up at 12 months and 24 months at 10% loss to follow-up for each visit, we found no substantial change in cervical cancer deaths or pre-cancer treatments compared to the base case assumption.

**Extended Data Fig. 3:**
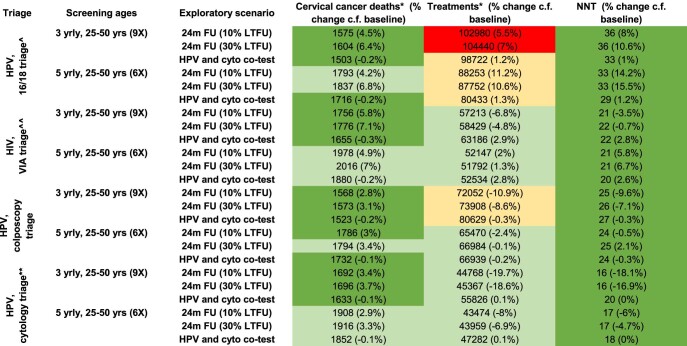
Summary of key outcomes for simulated screening approaches in supplementary analysis scenarios for post precancer treatment management. Number of predicted cervical cancer deaths, precancer treatments and the number needed to treat to prevent a cervical cancer death per 100,000 Tanzanian women living with HIV, compared to baseline and no screening scenarios for supplementary analyses considering the management of women following treatment for precancer. The range for the colour coding for each column is shown in Extended Data Fig. 1.

For women treated for cervical pre-cancer (without known CIN3+), the base case recommendation is for women to return in 12 months for follow-up testing with HPV. Relative to this base case assumption, supplementary analysis scenarios considering extending this interval to 24 months (30% loss to follow-up) resulted in an increase in expected cancer deaths by 3–10% and up to 18.6% fewer pre-cancer treatments (or 3–6% and up to 19.7%, respectively, if loss to follow-up was only 10%), depending on triaging strategy. If cytology and HPV co-testing replaced HPV as the follow-up test, we predicted a 0.1–0.4% reduction in cervical cancer deaths and up to 2.9% more pre-cancer treatments, relative to follow-up testing with HPV (Extended Data Fig. 3).

**Extended Data Fig. 4:**
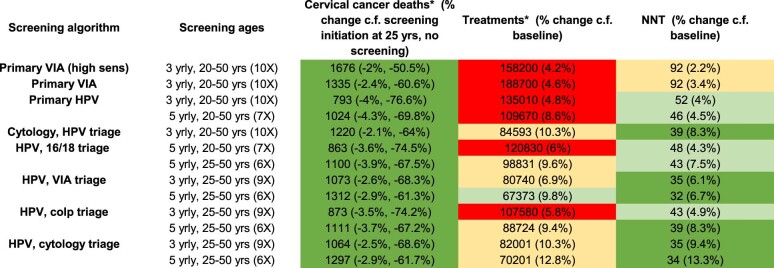
Summary of key outcomes for simulated screening approaches in supplementary analysis scenarios for an alternative screening start age. Number of predicted cervical cancer deaths, precancer treatments and the number needed to treat to prevent a cervical cancer death per 100,000 Tanzanian women living with HIV, compared to baseline and no screening scenarios for supplementary analyses considering a reduction in age of screening initiation to 20 years. The range for the colour coding for each column is shown in Extended Data Fig. 1.

We additionally considered the impact of bringing forward screening initiation to age 20 years. Here, we found that starting screening at age 20 years increased the number of lifetime cervical screening tests and reduced cancer deaths by up to 4.3% but increased pre-cancer treatments by 4.2–12.8%, relative to scenarios with screening initiation at age 25 years (Extended Data Fig. 4).

### Sensitivity analysis

In sensitivity analysis, we considered the impact of variation in screening adherence, primary test sensitivity and anti-retroviral therapy (ART) adherence and subsequent viral suppression among women living with HIV on primary simulated outcomes. In general, the simulated variation in these parameters did not impact the overall findings of this study—namely, that primary HPV testing is more effective than VIA or cytology at preventing cervical cancer incidence and death and that combining triage with primary HPV testing reduces harms associated with cervical screening.

**Extended Data Fig. 5:**
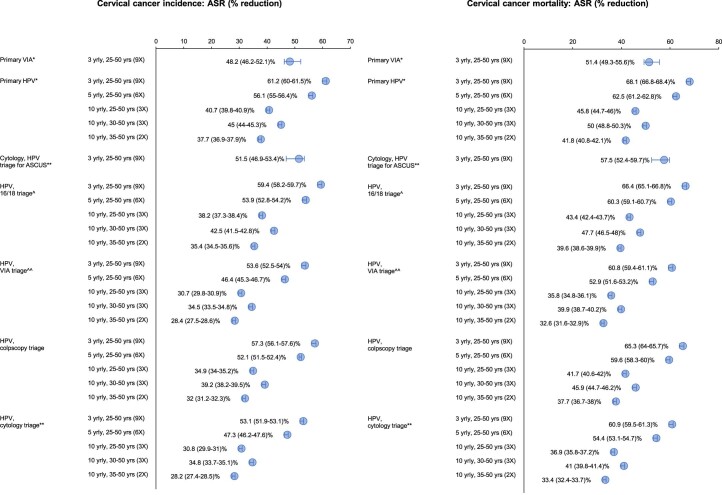
Effectiveness of simulated screening approaches considered in sensitivity analysis of screening adherence. Reductions in (**a**) ASIR and (**b**) ASMR compared to no screening in women living with HIV for sensitivity analysis considering reduced screening adherence. The error bars around the dots represent the reductions when assuming the best (upper range) and worst (lower range) primary test performance assumptions as described in the Methods. Age-standardisation is calculated using the 2015 World Female Population for ages 0–99 years.

Under base case compliance assumptions, we assumed that 10% of women do not ever attend for screening throughout their lifetime and that, of the remaining 90% of women, 70% comply with each recommended screening test; compliance to follow-up and post-treatment testing was assumed to be 90%. In sensitivity analysis, we considered a reduced compliance scenario, where 30% of women are assumed to not ever attend for screening, with the remaining 70% attending each recommended screening test 50% of the time; follow-up and post-treatment attendance was reduced to 70%. Under reduced compliance assumptions, primary HPV testing, with or without triage, at a screening interval of either 3 years or 5 years, was found to be most effective at reducing the age-standardized rates of cervical cancer incidence (46–66%) and mortality (54–72%) compared to no screening (Extended Data Fig. 5).

**Extended Data Fig. 6:**
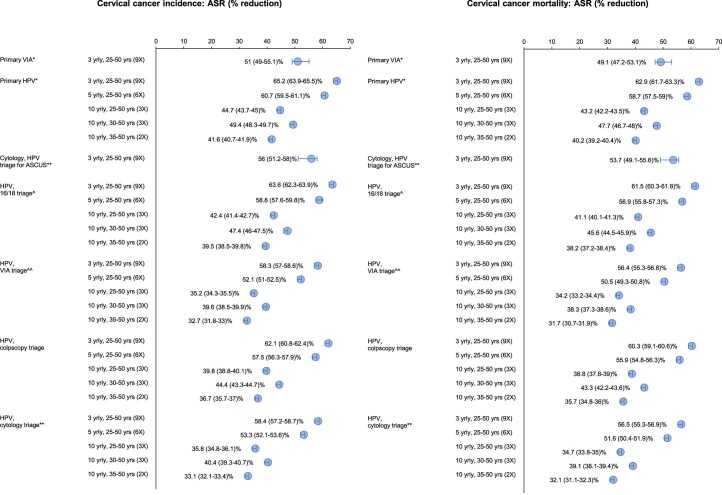
Effectiveness of simulated screening approaches considered in sensitivity analysis of cancer treatment access. Reductions in ASMR assuming (**a**) worst-case cancer treatment access and (**b**) best-case cancer treatment access compared to no screening in women living with HIV for sensitivity analysis. The error bars around the dots represent the reductions when assuming the best (upper range) and worst (lower range) primary test performance assumptions as described in the Methods. Age-standardisation is calculated using the 2015 World Female Population for ages 0–99 years.

Under base case cancer treatment assumptions, we assumed that 9.5% (ref. ^2^) of women with symptomatically detected cervical cancer and 90% of women with screen-detected cervical cancer receive appropriate cancer treatment. In sensitivity analysis, we considered ‘worst-case’ and ‘best-case’ cancer treatment scenarios whereby, regardless of detection modality (and, therefore, also for the ‘no screening’ scenario), the cervical cancer treatment rates are 9.5% and 90%, respectively (Extended Data Fig. 6).

**Extended Data Fig. 7:**
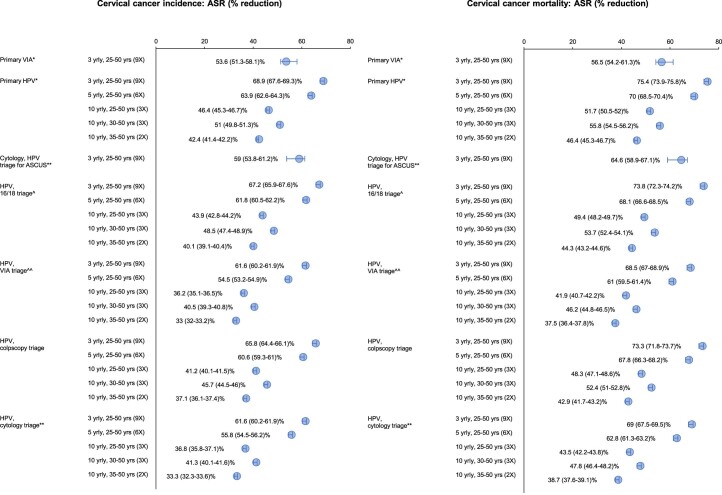
Effectiveness of simulated screening approaches considered in sensitivity analysis of HIV viral suppression. Reductions in (**a**) ASIR and (**b**) ASMR compared to no screening in women living with HIV for sensitivity analysis considering viral suppression rates. The error bars around the dots represent the reductions when assuming the best (upper range) and worst (lower range) primary test performance assumptions as described in Methods. Age-standardisation is calculated using the 2015 World Female Population for ages 0–99 years.

In sensitivity analysis, we considered a scenario where WHO 90–90–90 targets for HIV testing and treatment were achieved. In this scenario, there was increased benefit of cervical screening across all simulated screening approaches; however, the findings were consistent with the baseline findings (Extended Data Fig. 7).

## Discussion

We performed a modeled assessment of the benefits and harms of seven priority screening approaches in women living with HIV in Tanzania. These results formed part of the evidence base informing the development of updated WHO guidelines^8^, which recommend primary HPV testing with triage every 3 years or 5 years for women living with HIV aged 25–49 years.

Determining the optimal tests, algorithms and intervals for cervical screening and treatment is critical to inform global guidelines to reduce cervical cancer incidence and mortality and is a challenge for women living with HIV due to the paucity of longitudinal data. In our analysis, we assessed incidence, mortality and reproductive outcomes for 28 screening and treatment algorithms (considering variations in interval, primary test and triaging approach) in a setting with a high burden of both HIV and cervical cancer. Primary HPV without triage (‘screen-and-treat’) for women living with HIV aged 25–50 years was, by a small margin, the most effective approach for reducing cervical cancer incidence and mortality, with 3-yearly or 5-yearly testing reducing cervical cancer deaths by 67% and 63%, respectively, relative to no screening. However, approaches involving triaging HPV^+^ women before treatment resulted in minor loss to efficacy (1–5%) compared to HPV testing without triage with substantially reduced pre-cancer treatments (by 11–53%), thus improving the balance of benefits to harms (NNT). Compared to HPV-based screening, primary VIA and cytology were both less effective at 3-yearly screening intervals and were less efficient than primary HPV screening with triage. In our analysis for women living with HIV, we found that the effectiveness of frequent (3-yearly), high-quality cytology appears to approach 5-yearly HPV screening with triage. However, these findings assume high regular screening coverage and quality-controlled pathology, and, in practice, this would be difficult to achieve in LMIC settings.

In the companion paper, the benefits, harms and cost-effectiveness of screening approaches are reported for a general population of women living in 78 LMICs. Taking these papers together, we note that 3-yearly HPV screening with triage for women living with HIV is similar to 5-yearly HPV screening for women in the general population, achieving similar reductions in cervical cancer burden and similar efficiency (NNT of 20–33 versus 26–37).

In the current analysis, we focused on women living with HIV who had not been vaccinated as adolescents. Although the risk of cervical cancer at a population level is expected to reduce over time due to vaccination and HIV control, even under a best-case scenario of 90% of all eligible females in LMICs vaccinated by 2030, the full impact on cervical cancer incidence will take decades to realize. Thus, this analysis of the relative benefits and harms of various screening approaches in an unvaccinated cohort of mid-adult and older women will be broadly applicable at a population level for at least 10–20 years into the future. Future analyses may consider optimal screening in vaccinated populations, and it is important to note that the guidelines have been considered to be ‘living guidelines’, which can be updated in response to population changes in risk, the emergence of future technological developments or other factors.

In the current analysis, we used the setting of Tanzania as an exemplar. Tanzania carries a high burden of cervical cancer, with an ASIR of 62.5 in 2020, noting that women living with HIV account for 6.2% of the female population aged 15–49 years^15,16^. To address this burden, the revised National Cervical Cancer Prevention and Control Strategic Plan 2020–2024 in Tanzania outlines priorities toward both cervical cancer elimination and the United Nations Sustainable Development Goals, including bolstering the previously low (6–21% (refs. ^17,18^)) rates of screening participation under an HPV-based screening program^19^. This plan aligns with the National Cancer Control Strategy^20^, which focuses on unifying a national plan for cervical cancer control, advocating for funding and resources and setting a framework for monitoring key program indicators^19^. Although a transition to primary HPV testing is underway, Tanzania has previously implemented cervical screening with VIA and is not alone among many LMICs that have done so; it is, therefore, important to consider the role of VIA as a triage for women who screen positive for HPV, which, as this analysis has demonstrated, will not substantially lower the overall effectiveness of an HPV-based screening program if effective follow-up is in place for HPV-positive, VIA triage-negative women. Re-positioning VIA from a primary to an HPV triage test will support substantially more effective screening but will also leverage existing knowledge and infrastructure for VIA.

Using radiotherapy access as a proxy for access to any cancer treatment, we estimate that 9.5% of women diagnosed with cervical cancer in Tanzania currently have access to appropriate cancer treatment^2^, underscoring the importance of preventing cervical cancer where possible while investing in greater overall access to treatment services. Previous modeling analyses for Tanzania using the Policy1-Cervix-HIV platform quantified the impact of endemic HIV and HIV control on cervical cancer over time^13^, assessing the feasibility and timeliness of cervical cancer elimination under the WHO triple-intervention 90–70–90 targets in Tanzania^14^. These analyses found that endemic HIV itself has increased cervical cancer incidence rates in Tanzania, but methods for HIV control, including voluntary medical male circumcision (VMMC) and ART, have acted to reduce the burden of both HIV and cervical cancer^13^. Furthermore, achieving WHO targets for HPV vaccination coverage and participation in twice-lifetime HPV testing, with intensive 3-yearly screening for women living with HIV, could accelerate the elimination of cervical cancer in Tanzania to 2076 (ref. ^14^).

Many modeled parameters, including viral suppression rates, cancer treatment access and sexual behaviors, are reflective of Tanzania. In practice, these parameters would vary among countries, resulting in changes in overall cervical cancer burden at the population level. The impact of this variation on the relative differences between screening approaches is likely to be small; nonetheless, it would be optimal for individual countries to tailor their screening approaches for women living with HIV based on their local epidemiology. To assess the impact of using Tanzania-specific parameters, including viral suppression and cancer treatment access, and to assess the robustness of our findings to changes in screening test performance and attendance assumptions, we performed a range of univariate sensitivity analyses. These analyses demonstrated that, for all parameter combinations, HPV-based screening every 3 years regardless of triaging approach remains the most effective strategy.

A strength of our analyses is that modeling was performed in close consultation with the WHO Guidelines Development Group, and decisions on assumed screening test performance, scenarios and other key assumptions were determined in consultation with that group. For women living with HIV, we used Policy1-Cervix-HIV, an extensively validated model parameterized to Tanzania^13,14^, which was ideally positioned to simulate outcomes explicitly in a cohort of women living with HIV. Policy1-Cervix-HIV underwent further development to incorporate the detailed screening algorithms proposed by the Guidelines Development Group to assess the impact of using alternative screening technologies, intervals and methods for triage for women living with HIV. Using this independently developed platform in conjunction with the Policy1-Cervix platform used for the general population in 78 LMICs (the companion article) strengthens our combined assessment supporting the choice of primary HPV screening for all women, including women living with HIV.

Our analyses have several limitations, as, in many cases, our assumptions for this normative analysis may not reflect the reality of screening program implementation and uptake, particularly where services are limited. Baseline assumptions for routine screening participation of 70% (50% in sensitivity analysis) and follow-up attendance of 90% (50% in sensitivity analysis) represent a ‘realistic best-case’ scenario, as substantial investment will be required to achieve these targets. Due to the evidence available when the analysis was performed, we also assumed that excisional treatment success in women living with HIV is equivalent to the general population. Since that time, a systematic review and meta-analysis found that excisional treatment success is poorer in women living with HIV^21^; thus, our findings may reflect greater benefits for screen, triage and treat programs in women living with HIV than those actually achievable.

A further limitation is that we assumed that the probability of a pre-term delivery resulting from pre-cancer treatment and age-specific fertility rates are the same for women living with HIV as for the general population, an assumption based on a paucity of pregnancy outcome studies in women living with HIV. However, fertility rates in women living with HIV may be lower than women in the general population^22^, which would reduce the impact of pre-cancer treatments on additional pre-term deliveries in women living with HIV (Fig. 3). In addition to the above limitations, we considered women who acquire their HIV infection by age 25, earlier than the regional average^23^, which was necessary to ensure equitable comparisons between screening approaches and to determine whether screening is safe and effective for women at all levels of risk. Nonetheless, due to this assumption, our simulated outcomes may reflect a greater benefit of cervical screening than the average benefit experienced in the population. Although numerous one-way sensitivity analyses were conducted, probabilistic sensitivity analysis was not performed. However, individual impacts of each variable considered in sensitivity analysis did not alter the study outcome, nor did probabilistic sensitivity analysis alter the relative performance of interventions previously modeled by this platform^13,14^. Finally, for the current analysis, we focused on outcomes related to HPV and cervical cancer, but, given that HPV may also increase acquisition risk of HIV^24^, future analyses could also quantify the benefits of preventing and treating HPV infection in terms of reducing HIV burden.

The current analysis, the companion study and many previous evaluations^25–28^ found that primary HPV screening is more effective than other primary screening approaches when multiple rounds of screening over a lifetime are simulated. However, such a transition will require a substantial shift in practice in many LMICs. Anticipating these challenges, the updated WHO guidelines suggest continuing existing screening strategies until high-performance testing algorithms are in place^8,29^. Integration of cervical screening into existing HIV monitoring and treatment programs for women living with HIV is one promising approach to reaching women living with HIV for cervical screening^29,30^. There appears to be a high level of acceptability of cervical screening among women living in LMICs, but screening uptake is currently limited by financial and access constraints^31,32^. Successful implementation of specific strategies tailored to vulnerable populations will be critical not just in terms of addressing the substantial burden of disease in women living with HIV but also of promoting equity of outcomes across all groups of women. The development of practical and effective programmatic models of HPV screen, triage and treat for women living with HIV will depend on the availability of affordable HPV and triage tests, appropriate linkages with reproductive and HIV services and effective registry mechanisms for recalling women for surveillance follow-up or referring them for further management. The WHO guidelines were updated in late-2021 to include guidance on the use of primary HPV mRNA testing in the general population of women, for which further modeling was performed using *Policy1-Cervix*. Given limited evidence on outcomes for mRNA testing in women living with HIV, no recommendations were made regarding use in this population. Guidelines on the use of dual-stain cytology as a triage are under development, and other emerging triage approaches will also be considered in subsequent iterations for the living guidelines^33^^,^^34^.

In conclusion, our findings demonstrate that primary HPV testing every 3 years or 5 years for women living with HIV aged 25–49 as part of a primary ‘screen, triage and treat’ approach resulted in the most efficient reductions in cervical cancer incidence and mortality and also reduced harms. These modeled findings formed part of the evidence base considered in the formulation of the WHO’s updated 2021 cervical screening and treatment guidelines. A range of systematic review, feasibility and acceptability studies also informed the final recommendations, which are that women living with HIV be screened with primary HPV testing in a screen, triage and treat approach starting at age 25 years with regular screening every 3–5 years.

## Methods

Modeling was performed with the platform *Policy1-Cervix*, which is a collection of tools, including a natural history model, that has been adapted for many different countries and contexts. For modeling cervical screening and treatment in women with HIV, we harnessed a previously developed and parameterized version, *Policy-Cervix-HIV*, for women with HIV in Tanzania^13,14^, a country with endemic HIV, very high annual age-standardized rates of cervical cancer incidence (ASIR 62.5/100,000) and mortality (ASMR 42.7/100,000) and very limited access to and uptake of cervical screening^15^. In the present analysis, we simulated cervical screening approaches throughout the lifetime of a specific cohort of 100,000 women in Tanzania who acquired an HIV infection on or before their 25^th^ birthday. Women with an HIV infection are included in this cohort irrespective of their status on the HIV testing and treatment cascade, which includes diagnosis of an HIV infection, and ART uptake with or without achievement of viral suppression. We consider outcomes in unvaccinated women only; although vaccination programs may be implemented by this time in many LMICs, females who would be targeted by vaccination programs between 2020 and 2029 will not be screen age eligible until approximately 2040, and, even after this time, most women within the screening age ranges (30–49 years) will be unvaccinated for at least a few more decades.

Using Policy1-Cervix-HIV, we simulated a range of scenarios including a comparator, with no screening, no vaccination and no scale-up of cervical cancer treatment access, and a number of screening approach scenarios which feature a combination of screening age range, frequency and test technology informed by consultation with the WHO Guidelines Development Group; this process is described in the next subsection.

The Policy1-Cervix-HIV model is a deterministic transmission-dynamic compartment model of sexual behavior, HIV and HPV infection and natural history, which captures simultaneous HIV and HPV infections, including for multiple HPV types, and incorporates comprehensive demographic, sexual behavior and natural history assumptions by 5-year age groups. It also accounts for VMMC and ART for men and women living with HIV. The Policy1-Cervix-HIV model has been extensively calibrated to HIV prevalence, HPV prevalence and cervical cancer incidence and mortality observed in Tanzania, as described in detail in previous publications^13,14^ and in subsequent subsections. We report according to HPV-FRAME reporting standards for modeled evaluations of HPV prevention and control^35^. In this evaluation, Policy1-Cervix-HIV incorporates detailed cervical screening programs involving the management of screen-positive but triage-negative women and management of women after pre-cancer treatment. To report on additional pre-term deliveries due to pre-cancer treatments, a Monte Carlo simulation model was used that simulates adverse obstetric outcomes using country-specific and age-specific fertility and pre-cancer treatment rates.

### WHO guidelines development process

To inform the revision of the guidelines for cervical screening and pre-cancer treatment, the WHO convened an expert technical team that consisted of experts across a broad range of domains in cervical screening. Members of the team met regularly to progress the accumulation and synthesis of evidence informing the safety, effectiveness, cost implications and potential harms of priority cervical screening technologies and algorithms, and this group was involved in key decision-making about input assumptions for the modeling. Preliminary findings from the modeled analysis were presented on multiple occasions (on 28–29 July 2020 and 30 September 2020), and assumptions and interpretations were discussed. Here we report specifically on the modeling component of this evidence, informing recommendations for women with HIV; the modeled evidence informing recommendations for the general population of women is reported elsewhere^36^.

The WHO Guidelines Development Group identified 7 priority algorithms that would be potentially suitable for LMICs, including primary VIA, primary cytology with HPV triage (ASC-US referral), primary HPV without triage (all HPV^+^ women treated after using assessment of eligibility for ablative treatment), primary HPV 16/18 triage, primary HPV VIA triage, primary HPV cytology triage and primary HPV colposcopy triage. To ensure adequate communication among the different expert groups involved in informing the update of cervical screening guidelines, weekly meetings were held among the modeling team, representatives from the WHO secretariat and representatives from the systematic review and costing teams. Regular meetings were also held between Guidelines Development Group members and the systematic review, modeling and costing teams to discuss the priority management algorithms. The modeled evaluation was performed over a 3-stage process. In the first stage, we evaluated the benefits and harms (using pre-cancer treatments as a proxy for harms) of the 7 priority algorithms, considering various screening ages and frequencies. These results were presented to the Guidelines Development Group in July 2020. In the second stage, we included results on additional adverse obstetric outcomes as a result of pre-cancer treatments as a measure of the harms associated with screening, as well as cost-effectiveness outcomes for women in the general population, and presented these to the Guidelines Development Group in September 2020. The third stage involved a detailed exploration of the optimal management of women after negative triage test and the optimal management of women after treatment for pre-cancer; modeled evaluations of these alternative management options were presented to the Guidelines Development Group in November 2020.

### Scenarios for evaluation

In the base case analysis, we simulated seven priority screening algorithms as identified by the Guidelines Development Group: primary VIA, primary HPV (no triage but with VIA to determine treatment eligibility), primary cytology with HPV triage in case of finding ASC-US cytology, primary HPV with HPV 16/18 triage, primary HPV with VIA triage, primary HPV with cytology triage and primary HPV with colposcopy triage (Table 1). These priority algorithms were selected by the Guidelines Development Group for consideration due to the availability of quality data on test sensitivity and specificity as well as evaluations of the potential feasibility, costs and acceptability of possible implementation. Notably, detailed management for each of these screening scenarios, including downstream management for women in follow-up, at colposcopy and after pre-cancer treatment, is described by the WHO online^36^. Variations in age ranges and screening frequencies considered for women with HIV generated a total of 27 scenarios, which were then modeled (Table 1). In this analysis, a driving reason for choosing an end age of 50 years was the higher rate of comorbidities in women aged over 50 years and the lower life expectancy across LMICs, thereby reducing the amount of disability-free life-years that could be gained by screening these older populations. Additionally, inadequate visualization of the transformation zone is typical after menopause, and ablative treatment is not suitable for treatment in situations in which the transformation zone is not visible. As countries develop and life expectancies increase, the age to end screening could be adjusted to account for increasing life expectancy and improved quality of life in these age groups.

The simulated intervention scenarios are compared to a ‘no screening’ comparator rather than current screening practices in Tanzania (6–21% of women ever screened with VIA^17,18^). The rationale for this is twofold: first, the specific conditions of cervical screening in Tanzania are not generalizable to other settings with endemic HIV; second, the impact of VIA screening on cervical cancer incidence is limited, and multiple rounds of screening are required to demonstrate downstaging and mortality benefit^37,38^.

For all simulated screening algorithms, we considered an unvaccinated cohort of women with HIV who were born in 2005 and who acquired HIV by age 25 years. This cohort of women was chosen because they would be the first to benefit from the new screening recommendations over their lifetime, assuming full program implementation by 2030. This cohort of women with HIV is assumed to uptake and adhere to ART throughout the course of their illness, such that population-level HIV plasma viral suppression rates of 47% among all women living with HIV are maintained, for consistency with 2018 UNAIDS data for viral suppression in Tanzania^39^; further details on viral suppression by age are contained in later subsections on model parameterization.

**Extended Data Fig. 8:**
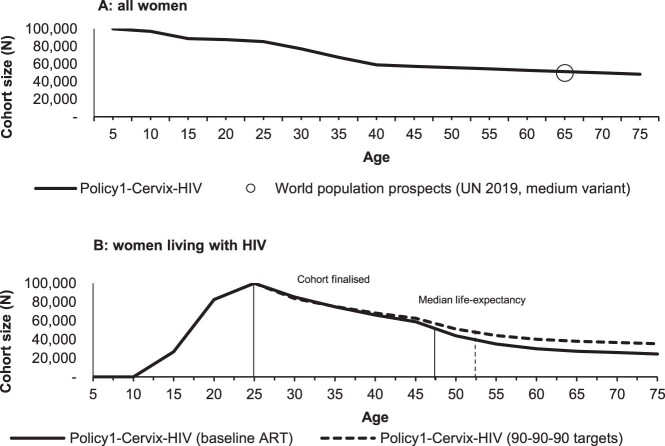
Simulated cohort characteristics. Simulated cohort size of 100,000 Tanzanian women born in 2005 in (**A**) the general population, and (**B**) the population subgroup who acquire HIV on or before their 25^th^ birthday (base-case ART assumptions compared to WHO 90–90–90 ART assumptions).

The simulated cohort is at higher risk than women in the general population of all-cause mortality, as demonstrated by a difference in life expectancy of over 25 years (Extended Data Fig. 8). Notably, increasing rates of viral suppression to meeting the UNAIDS 90–90–90 targets for HIV testing and treatment is predicted to increase life expectancy by 10 years in this cohort.

**Extended Data Fig. 9:**
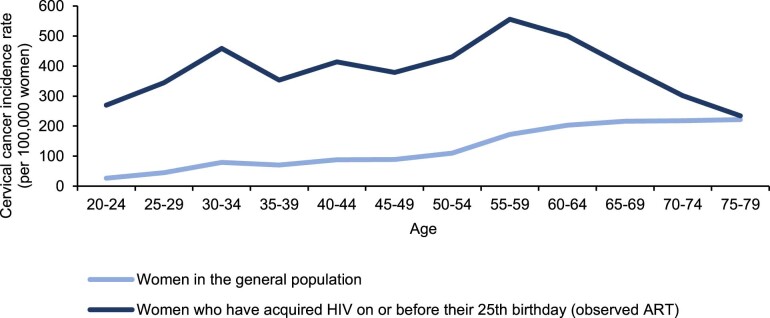
Age-specific cervical cancer incidence in the simulated cohort. Simulated age-specific cervical cancer incidence among women who acquire HIV on/before their 25^th^ birthday, versus women in the general population born in 2005.

Furthermore, this subgroup of women is subject to substantially increased risk of cervical cancer incidence with an age-weighted relative risk (RR) of cervical cancer of 5.3, with the increase in risk being heavily weighted toward women at younger ages despite high rates of viral suppression (Extended Data Fig. 9).

### Simulation outcomes

Simulated outcomes included the average estimated lifetime number of cervical cancer cases and deaths and ablative and excisional pre-cancer treatments per woman. We additionally calculated age-standardized rates of cervical cancer incidence and mortality, the number of pre-cancer treatments and screening tests required to prevent a cervical cancer death (NNT and NNS) and the number of additional pre-term deliveries due to pre-cancer treatment. Reductions in cervical cancer incidence and mortality, rates and overall numbers are considered benefits of screening, whereas pre-cancer treatment and any resultant pre-term deliveries are considered screening harms.

### Primary test characteristics

Model assumptions for primary test characteristics were based on an updated systematic review that was conducted to inform the WHO Guidelines Development Group on cross-sectional sensitivity and specificity of a range of screening and triage tests for both general women and women living with HIV. The updated review did not include primary HPV (without triage) or primary cytology performance, and so published systematic review evidence was used for these tests. A detailed explanation describing all test sensitivity and specificity assumptions, including a review of evidence underlying these assumptions, is contained within the online methods of the companion to this article (Simms and Keane et al.). Based on this review, we developed a set of assumptions, for women in the general population and women living with HIV alike, which appears as Table 3 in the online methods to our companion article (Simms and Keane et al.). Among women living with HIV, these test positivity rates translate to slightly different sensitivity and specificity due to differences in underlying health state distribution. In particular, model-calculated sensitivity and specificity at a CIN2+ threshold for the simulated cohort of women with HIV is 96–98% sensitivity and 54–97% specificity for primary HPV; 38–46% sensitivity and 69–71% specificity for primary VIA; and 71–83% sensitivity and 67–75% specificity (or 57–72% sensitivity and 97% specificity at an low-grade squamous intraepithelial lesion (LSIL) threshold) for primary cytology. These assumptions are broadly consistent with the findings of an updated systematic review and meta-analysis of screening test performance in women with HIV^4^. This review reported a high degree of heterogeneity among studies estimating primary test characteristics, where, across all included studies, primary HPV had a sensitivity of 85.7–100.0% and a specificity of 41.3–77.4%; primary VIA had a sensitivity of 43.8–86.6% and a specificity of 47.3–96.7%; and primary cytology had a sensitivity of 20.0–78.4%.

Without modifying the health-state-specific test positivity rates for women living with HIV compared to women in the general population, we achieved similar trends in relative test characteristics as observed in the updated systematic review and meta-analsyis (Kelly et al., Diagnostic accuracy of cervical cancer screening strategies for high-grade cervical intraepithelial neoplasia among women living with HIV: a systematic review and meta-analysis (unpublished)). Overall, Kelly et al. reported that, compared to women in the general population, HPV test specificity for women with HIV was substantially (~33%) lower; however, this effect was reduced for women with well-controlled HIV (ART adherent for >2 years). Furthermore, although evidence for relative test performance of primary VIA for women living with HIV compared to women in the general population was weak, the review additionally noted that a lower sensitivity at CIN2+ was observed for women in the general population and that primary cytology test characteristics were largely similar, which is consistent with modeled test characteristics. In this way, we validated our existing input assumptions for primary test positivity for women living with HIV by comparison to this updated systematic review.

### Screening attendance and treatment delivery for cervical pre-cancer and cancer

For the base case analysis, we assumed that 10% of women would never attend screening and that 70% of all women would attend each routine screening visit (selected from the 90% of ever-screeners). We assumed that women referred for follow-up or treatment would attend at 90% adherence or 100% adherence if treatment could be offered on the same day after primary VIA testing. For HPV screen-and-treat scenarios, we assumed (for simplicity) that an HPV point-of-care test and same-day treatment could be conducted 50% of the time, and, therefore, 95% of women requiring treatment would be treated overall. Women referred for excision or further evaluation and workup for a diagnosis of cervical cancer were assumed to attend such visits with 90% adherence. Women who do not attend follow-up or treatment are assumed to have no further intervention for the screening round but are modeled as attending their next screening event (at 70% compliance) unless they are beyond the screening age recommendation. We assumed that 90% of screen-detected cervical cancer cases receive cervical cancer treatment or palliative care; however, the proportion of women who would receive cervical cancer treatment after diagnosis by symptomatic detection was assumed to be 9.5%, which is consistent with observed current cervical cancer treatment access in Tanzania^2^.

For invasive cancers, all scenarios will assume invasive cervical cancer clinical staging according to the International Federation of Gynecology and Obstetrics (FIGO) system. Stage distribution at detection was provided by the WHO for the earlier elimination analysis performed as part of the CCEMC^2^.

All screening scenarios assumed that 90% of screen-detected cancers would receive adequate treatment and care, and, therefore, we assumed improved survival for screen-detected cancers. Due to the added impact of potential down-staging, the relative survival for screen-detected cervical cancer compared to symptomatically detected cervical cancer is additionally assumed to be scaled up by 1.15– 1.17, depending on disease stage, regardless of whether the woman received adequate treatment and care^40–42^, as described previously^2^.

Under advisement by the Guidelines Development Group, we chose assumptions representing a ‘realistic best-case scenario’, understanding that participation is unlikely to be this high in all settings. However, we note that these targets are consistent with the WHO’s Global Strategy for the elimination of cervical cancer, which was endorsed by all member states back in 2020.

### Supplementary analyses

Several additional supplementary scenarios were considered in stage 3 analysis to evaluate several possible primary screening follow-up recommendation options for women with HIV. These included starting screening at a younger age, alternative follow-up management for screen-positive women who are triage test negative and alternate management options after pre-cancer treatment.

#### Management of HPV^+^ women

In supplementary analysis, we considered variation in the management of women who tested positive at their primary HPV test but were determined to be at low/intermediate risk of CIN3+ after triaging. Under base case assumptions, triage-negative women were recommended to return for follow-up HPV testing at 12 months after the initial test and, depending on the result of this follow-up test, would either receive pre-cancer treatment or be discharged to routine screening. We then additionally considered three management pathway scenarios for these women where (1) the follow-up interval is extended to 24 months (10% loss to follow-up), (2) follow-up interval is extended to 24 months (30% loss to follow-up) and (3) an additional round of follow-up was considered where follow-up tests are recommended at 12 months and 24 months after the initial test, each with 10% loss to follow-up.

#### Management of women after pre-cancer treatment

We considered alternative assumptions for the follow-up of women treated for cervical pre-cancer. Under base case assumptions, women who have undergone pre-cancer treatment who had histologically confirmed CIN3+ are referred for repeat HPV testing at 12 months and 24 months. All other women (that is, women without any histologically confirmed CIN3+) who underwent pre-cancer treatment are followed-up with HPV testing at 12 months. In supplementary analysis, for women without histologically confirmed CIN3+, we considered extending this follow-up interval to 24 months (assuming loss to follow-up of 10% and 30%) in addition to a more aggressive management option where women are followed up at 12 months with co-testing (both HPV and cytology) at 10% loss to follow-up.

#### Screening start age

In an additional screening sub-analysis, we considered the impact of reducing the screening start age from 25 years (for strategies with this start age in the base case) to 20 years, for 3-yearly and 5-yearly intervals and for all test technologies. In this sub-analysis, we considered a different cohort of Tanzanian women, born in 2010, who become infected with HIV before screening initiation at age 20 years. This alternative cohort was chosen because they are the first cohort who will potentially benefit from a lifetime of cervical screening from the age of 20 years.

### Sensitivity analysis

We performed a range of one-way sensitivity analyses, which included simulating ranges in screening and follow-up adherence, test performance characteristics, cervical cancer treatment and survival and ART treatment uptake and viral suppression.

#### Screening adherence

For all scenarios, we assessed the impact of lower screening adherence, assuming 50% adherence with routine attendance (30% of women never attend, 50% selected from the pool of ever-screeners) and 75% for adherence with treatment or follow-up visits (100% for same-day eligibility or 87.5% for potential same-day eligibility after a HPV^+^ test). For supplementary scenarios with follow-up testing at 24 months, we additionally considered loss-to-follow-up rates of 30% (compared to the 10% base case assumption), as described above.

#### Test performance characteristics

We considered variations in CIN2+ sensitivity for primary screening tests, including primary VIA (lower bound = 30.0%, upper bound = 60.0%), primary HPV (lower bound = 88.0%, upper bound = 95.7%) and primary cytology with LSIL threshold (lower bound = 46.8%, upper bound = 80.0%).

#### ART and HIV suppression

We assessed the impact of an alternative (upper bound) HIV control scenario, in which Tanzania meets the UNAIDS 90–90–90 targets for HIV testing, treatment and control by 2030 (ref. ^43^). For this assessment, we assumed that, at HIV diagnosis, 73% of all women living with HIV are recruited into ART programs and achieve and maintain viral suppression throughout the course of their life, compared to the 47% assumed in base case scenarios. The remainder of women are assumed to either never initiate ART or have incomplete viral suppression that partially protects against HIV death only.

### Model platform and data sources

#### Demographic characteristics

In the Policy1-Cervix-HIV platform, population size and structure are simulated by the demography module, which governs fertility and natural mortality. Births are simulated by assuming an age-specific fertility rate for women of childbearing age, which is calculated from observed year-on-year population-level fertility rates and data on maternal age at birth^44,45^. The age distribution of the starting population is based on the 1960 Tanzanian population^46^, and a sex ratio of 1 male to 1.03 females is applied to births^47^.

The simulated population is subject to an age-specific probability of death resulting from any cause other than HIV or cervical cancer (other-cause mortality). Age-specific and year-specific mortality rates are specified using the projected year-on-year life tables reported by the United Nations Population Division^48^.

The distribution of behavioral characteristics related to sexual risk-taking is simulated along with population demography. Annually, the model checks that the proportion of men and women sitting in ‘high-activity’ compartments versus ‘general-activity’ compartments, as well as the recruitment and retirement of women into or out of commercial sex work, is appropriate for each age group. If there is found to be an imbalance in these ratios, males and females are redistributed proportionally to their other characteristics.

#### Sexual behavior and force of infection

Males and females in the model are distributed into risk-based groups associated with sexual activity; these are ‘high activity’ and ‘general activity’. The assumed sex-specific, age-specific and year-specific proportions of simulated individuals being in each activity group, and the age-specific rate of partnership turnover for each group, were found by taking the findings from a sexual behavior survey of randomly sampled adults across rural Tanzanian communities^49^ together with known HIV and HPV transmission factors and prevalence.

Furthermore, the model explicitely accounts for sex work. Based on data from the UNAIDS 2018 update, we assume that 1% of females aged 15–54 years in Tanzania are sex workers^39^. Among the population of female sex workers, we assume that 36% are aged 15–24 years, 40% are aged 25–34 years, 22% are aged 35–49 years and 2% are aged 50–54 years, which was informed by a National Advisory Council on Poverty report on female sex workers in Dar es Salaam (2010)^50^. The average number of clients per sex worker is assumed to be 26 per month based on a survey of women engaged in sex work and transactional sex in Tanzania and is weighted by the age distribution of sex workers^50,51^. The age-specific rate of males interacting with female sex workers matches the observed probability of men having paid for sex in the prior 12 months, published in the 2007–2008 HIV/AIDS and Malaria Indicator Survey^52^.

Uptake of preventative interventions, such as condom use, VMMC and ART adherence, was input directly based on observed data. Modeled average condom use in sexual interactions is scaled linearly among three data points—<5% in 1993, 55% in 2011 and 37% in 2016—which matches survey responses in non-commercial sex interactions^52–57^. VMMC prevalence among sexually active males is specified by year and is specified to match observed Tanzanian data^56,58^. We assume an 8% prevalence of VMMC (or traditional circumcision where applicable) until 1995. From 1996, VMMC prevalenc increased linearly to 23% in 1998 and then increased quadratically to 80% in 2015. Viral suppression rates due to ART uptake and adherence have also been specified to match observed Tanzanian data and are based on UNAIDS estimates of the HIV testing and treatment cascade^59^. We assume ART programs are introduced in 2005, with viral suppression rates increasing linearly over 2005–2017 to reach 47%. Women who are HIV^+^, know their status and are receiving treatment but not virally suppressed are also simulated (incomplete suppression).

#### Natural history of HPV and HIV

Males and females with an HIV infection are assumed to progress linearly through disease stages aligning with the WHO Clinical Staging of HIV/AIDS for Adults and Adolescents^60^. These stages are acute infection, followed by WHO clinical stages 1 through 4, with the average timeframe from HIV acquisition to AIDS mortality for untreated individuals being 10–11 years^61^.

HIV infection dynamics and state transition probabilities are assumed to occur independently of the presence of any HPV infection; however, HIV disease stage and treatment status are assumed to directly influence state transition probabilities governing HPV natural history.

HPV progression and regression are governed by assuming age-specific, HPV-type-specific and HIV-status-specific progression and regression probabilities through HPV negative (immune, susceptible), pre-cancerous (prevalent HPV, productive HPV infection with CIN1, CIN2, CIN3) and cancerous (undetected and detected cancer for FIGO 1a–4b) states. The natural history of HPV infection is explicitly simulated for HPV types 16 and 18, the HPV types included in the nine-valent vaccine (31/33/45/52/58 abbreviated as HPV H5), and other oncogenic HPV types (abbreviated as HPV OHR). Cervical cancer survival is simulated, independent of HPV genotype, after the detection of cervical cancer. For each (detected) cervical cancer stage, the assumed probability of survival is 0.083, 0.064 and 0.009 for FIGO stages 1, 2/3 and 4, respectively. These survival probabilities are based on 10-year survival probabilities estimated for Tanzania^2^.

Women who are HIV^+^ are at greater risk of cervical cancer than HIV^−^ women and experience higher rates of HPV disease progression and lower rates of HPV disease regression.

HIV positivity status and viral suppression through ART are both assumed to affect HPV acquisition and natural history; assumptions regarding the impact of HIV positivity on HPV natural history are summarized in Supplementary Table 1.

Viral suppression due to ART is assumed to return HPV disease progression risk to near HPV^−^ levels (95% return to base transition probability values).

#### HIV control interventions

The model platform incorporates a range of control interventions for HIV, including ART for HIV modifying HIV disease natural history and reducing transmissibility; VMMC, which reduces the risk of both HIV and HPV acquisition in males; and pre-exposure prophylaxis (PrEP) for HIV prevention of HIV acquisition. These interventions are described in Supplementary Table 2.

### Model calibration and validation outcomes

Details of the model parametrization process and calibrated model variables presented here have been adapted and reproduced from a previous publication^13^ and the open-access doctoral thesis from which this study forms a chapter^62^. The input parameters are specified primarily using empirical data; however, some parameters, if unobservable or informed by survey data, were found through calibration using MATLAB’s (R2018b) inbuilt nonlinear least squares solver for data fitting called ‘lsqnonlin’, which used a trust region reflective algorithm option. The calibrated inputs included sex-specific, age-specific and activity-group-specific volumes of high-risk sexual contacts per timestep, the degree of age-assortative sexual mixing, annual fluctuations in population-level risk-aversive behavior and the relative per-sex-act probability of HIV acquisition for females compared to males. These inputs were calibrated to estimated HIV prevalence over time and stratified sex and annual rates of new HIV infections obtained from UNAIDS^63–65^.

A complete description of the parameterized model fit to observed data from Tanzania appear in refs. ^13^^,14^.

Simulations from the calibrated model were consistent with observed HIV-specific outcomes from UNAIDS, including male and female HIV prevalence, total HIV incidence and number of HIV deaths (see Fig. 3 in Hall et al.^13^)^39,63–65^. A robust fit was achieved for a range of calibration targets, with the model predicting lower HIV prevalence among males than the observed data for some years; given the strong fit to HIV prevalence among simulated females, it is unlikely that this will impact simulated outcomes in the previous or current analysis. Calibrated model outcomes were additionally similar to age-specific cervical cancer incidence and mortality rates for 2018 as reported by the International Agency for Research on Cancer^66^ and are reported in Fig. 4 of Hall et al.^13^.

Following the calibration procedure, the model was validated against independent data (that is, not used in model training). For HIV-specific outcomes, validation data included sex-specific and age-specific HIV prevalence and the sex-specific age distribution of age at AIDS diagnosis. The model fit to these data appears as Fig. 5 in Hall et al.^13^, and the underlying validation data were sourced from the Tanzanian Ministry of Finance.

Validation data for HPV and cervical cancer-related outcomes included age-specific HPV prevalence (Fig. 6 in Hall et al.^13^) and the prevalence of high-grade squamous intra-epithelial lesion (HSIL), considered equivalent to a diagnosed CIN 2/3 prevalence among HIV^−^ versus HIV^+^ women (Fig. 7 in Hall et al.^13^) from the PROTECT study^67^.

We additionally validated our model against published estimates reporting that women with HIV are at a 6.07 increased RR of cervical cancer^3^. To better reflect the study population, we compared this increase in risk to model-predicted cervical cancer incidence rates in Tanzania in 2020. More specifically, we predict that age-standardized cervical cancer incidence rates in women with HIV in Tanzania were 6.7-fold higher than their peers without an HIV infection and 4.4-fold higher than rates across the entire population.

**Extended Data Fig. 10:**
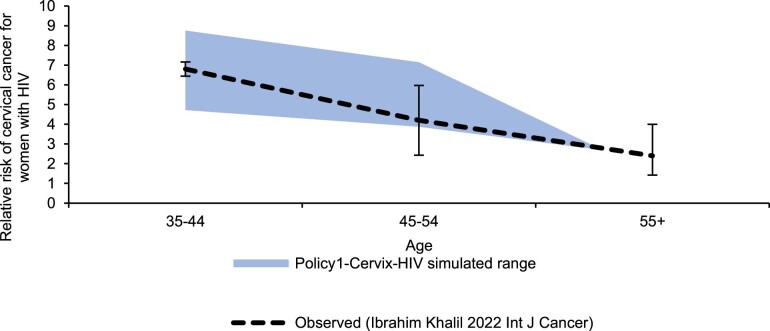
Model validation to observed age-specific cervical cancer risk in women living with HIV. Age-specific relative risk of cervical cancer in women with HIV compared as simulated by Policy1-Cervix-HIV for the current analysis (2005 birth cohort acquiring HIV by age 25) compared to published observed rates (ranges reported in the observed data represent 95% confidence intervals).

Additionally, our simulation outcome for the current analysis in a cohort of women born in 2005 who acquire HIV by age 25 years compares well against estimates of the age-specific increase in cervical cancer risk due to HIV (Extended Data Fig. 10)^68^.

### Model of obstetric complications

To evaluate adverse obstetric outcomes due to pre-cancer treatment, we developed a Monte Carlo individual-based simulation model that incorporates country-specific and age-specific fertility rates, as well as pre-cancer treatment outcomes by mode of treatment, and explicitly models additional pre-term delivery events as a result of pre-cancer treatments in Tanzania. We obtained national age-specific fertility rates for Tanzania from the United Nations (2019)^69^. Predictions of age-specific treatment rates, by treatment type, were input to a model of obstetric outcomes developed for *Policy1-Cervix*, which incorporates systematic review data indicating that the risk of pre-term delivery after excision is higher than the risk after ablation (excision versus no treatment: 11.2% versus 5.5%, RR = 1.87, 95% confidence interval (CI): 1.64–2.12; ablation versus no treatment: 7.7% versus 4.6%, RR = 1.35, 95% CI: 1.20–1.52)^70^. We assumed that multiple treatments of the same type do not generate any additional risk of adverse pregnancy outcomes. We also considered a scenario in which ablative treatments did not increase the probability of pre-term deliveries for subsequent pregnancies.

### Statistical analysis

This study does not include a statistical analysis component.

### HPV-FRAME reporting standard for women living with HIV

The checklist below includes core reporting standard (Supplementary Tables 3 and 4), reporting standard for model of HPV vaccination, model of integrated HPV vaccination and cervical screening and model for LMICs, according to Canfell et al.^35^.

### Ethics and inclusion statement

This paper is one of a pair of papers to inform the updated WHO 2021 guidelines for screening and pre-cancer treatment for cervical cancer prevention—one for the general population and the current paper for women living with HIV. This research was conducted in close collaboration with the WHO Guidelines Development Group for Screening and Treatment to Prevent Cervical Cancer, which comprises a range of scientists, healthcare providers, implementers, ministry of health representatives, systematic reviewers, program implementation experts and representatives from civil society. The Guidelines Development Group comprises members from all five WHO regions (AFRO, SEARO, WPRO, EURO and EMRO) and, using the GRADE framework and the WHO Handbook for Guideline Development, assessed cervical screening options with a focus on LMICs, including countries with high HIV prevalence. The use of Tanzania-specific modeling for women living with HIV was conducted in collaboration with local co-authors and cites local published research, including current epidemiologic metrics for both HPV and HIV disease and cervical cancer prevention, including HPV vaccination and cervical screening and treatment.

### Reporting summary

Further information on research design is available in the Nature Portfolio Reporting Summary linked to this article.

## Online content

Any methods, additional references, Nature Portfolio reporting summaries, source data, extended data, supplementary information, acknowledgements, peer review information; details of author contributions and competing interests; and statements of data and code availability are available at 10.1038/s41591-023-02601-3.

### Supplementary information

Supplementary Information Supplementary Tables 1–4 with captions.

Reporting Summary

## Figures and Tables

**Extended Data Fig. 1:**
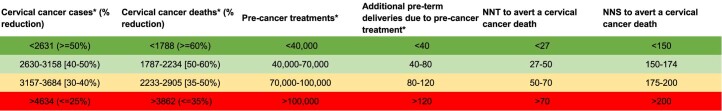
Numerical definitions for colouring of reported simulated outcomes. Numerical definitions for colouring of reported simulated outcomes specific to Fig. 1. Cells are coloured to provide an overall impression of strategies that are performing well – best performing strategies in a column are coloured green (best = largest cancer incidence/mortality reduction, or the lowest number of precancer treatments, NNTs or costs), followed by teal, yellow and then red for the worst performing strategies.

## Data Availability

Data on test performance, screening algorithms and compliance assumptions relevant to this specific evaluation are described in Methods. Demographic data and data informing the calibration to HIV-specific and HPV-specific targets in Tanzania are described in detail in Methods and in previous publications^13,14^.

## Code availability

The model used for this evaluation, Policy1-Cervix-HIV, is a well-established model platform spanning multiple software programs and related tools that has been developed over a period of 20 years. The code for these software programs is propriety property. These software programs, modules and tools consist of multiple versions for use in different contexts, and their accurate and appropriate use requires substantial supervised training. For these reasons, the code cannot be provided universally by the authors at this time. We will consider collaborative opportunities harnessing the code, and interested parties are encouraged to contact the Daffodil Centre at info@daffodilcentre.org. Indicatively, discussions can be held with interested parties within 4–8 weeks of the request, and plans for work will be put in place after that time, if successful. Appropriately resourced supervision will be provided by Daffodil Centre staff.
