# Supplementary material for: Benefits and harms of cervical screening, triage and treatment strategies in women living with HIV
Source: Nat Med. 2023 Dec 12;29(12):3059–66. doi: 10.1038/s41591-023-02601-3 (PMC10719091; doi:10.1038/s41591-023-02601-3)
Supplement: Supplementary file 1 — Supplementary Tables 1–4 with captions. [file 41591_2023_2601_MOESM1_ESM.pdf]

nature medicine

Article

https://doi.org/10.1038/s41591-023-02601-3

Benefits and harms of cervical screening, triage and treatment strategies in women living with HIV

In the format provided by the authors and unedited

Supplementary Table 1 HPV-type specific multipliers for eventual risk of acquisition, progression and regression of HPV associated disease for HIV positive individuals[38]. Table reproduced with minor alterations from Hall et al. 2020 PLOS ONE.[1]*

**Parameter description**	**Assumed arameter value[+]**
Multiplier increasing HPV acquisition for HIV positive individuals	2.75
Clearance of an HPV infection (no CIN)	0.6
Progression from HPV infection to CIN1	3.73
Progression from HPV infection to CIN2	1.3
Regression from CIN1 to clearance or HPV infected	0.7 for HIV 16/18<br>0.67 for all other HPV types.
Regression from CIN2/3 to clearance, HPV infection or CIN1	0.57
Progression from CIN3 to invasive cervical cancer	2.5

[+]*Note that these parameters are the same for all HPV types except where otherwise stated.*

Supplementary Table 2 Summary of disease control interventions for HIV.

**Intervention**	**Disease**	**Description**
Anti-retroviral therapy (ART)	HIV (secondary benefit to HPV in HIV-positive women)	In virally suppressed patients, ART halts disease progression and reduces HIV-related mortality in patients without AIDS. HIV-positive women with HPV infection and/or cervical precancer experience a more aggressive HPV natural history, which can be mitigated through viral suppression. Partially treated individuals experience reduced HIV-mortality rates, with no benefit to cervical cancer risk.
Voluntary medical male circumcision (VMMC)	HIV and HPV	VMMC reduces the per-timestep risk of HIV and HPV acquisition in males.
Condom use	HIV and HPV	Condom use reduces the per-timestep risk of HIV and HPV acquisition in males and females.
Pre-exposure prophylaxis (PrEP)	HIV	PrEP is assumed to remove males and females from the population susceptible to HIV. Assumed coverage of PrEP in this analysis is 0%.

Supplementary Table 3 HPV-FRAME analysis domain descriptions.

Domain	Description
CRS	Core reporting standards
1	Vaccination in adolescents
2	Vaccination in adults
3	Vaccination in MSM
4	Models of HPV-associated cancers among individuals living with HIV (ILWH).
5	HPV prevention in LMIC
6	Cervical screening (including integrated vaccination and screening)
7	Alternative vaccine types or reduced-dose schedules

** screening should be included for countries where significant screening exists*

Supplementary Table 4 Reportable model input and output descriptions for this analysis.

Domain	Inputs	Reported by age? (Y/N)	Report by sex (F/M/Both)?	Comments
CRS	Target population for intervention	Y	F	Reported in methods sub-section titled 'Scenarios for evaluation'.
CRS	Sexual behaviour	Y	Both	Reported in previous publication (Hall et. al.PLoS ONE 2020).[1]
CRS	Cohort examined for evaluation/ time horizon	Y	F	Reported in methods sub-section titled 'Scenarios for evaluation'.
CRS	Quality of life assumptions	N/A	N/A	Not applicable.
CRS	Calibration	Y	Both	Reported in a previous publication (Hall et. al.PLoS ONE 2020).[1]
CRS	Validation (where possible)	Y	Both	Reported in a previous publication (Hall et. al.PLoS ONE 2020).[1]
CRS	Costs	N/A	N/A	Not applicable.
2	Vaccine coverage at older ages	N/A	N/A	Not applicable.
1	Vaccine uptake	N/A	N/A	Not applicable.
1	Vaccine efficacy	N/A	N/A	Not applicable.
1	Vaccine cross-protection	N/A	N/A	Not applicable.
1	Duration vaccine protection and waning	N/A	N/A	Not applicable.
1	Vaccine and delivery costs	N/A	N/A	Not applicable.

1	Pre-vaccination disease burden (including population attributable fractions for HPV)	Y	F	Reported in a previous publication (Hall et. al.PLoS ONE 2020).[1]
1	Heterogeneity in sexual behaviour	Y	Both	Reported in a previous publication (Hall et. al.PLoS ONE 2020).[1]
1	Duration of natural immunity	N/A	Both	Reported in a previous publication (Hall et. al.PLoS ONE 2020).[1]
2	Natural history parameters, specifically for older individuals: structure; rate of infection clearance; loss of natural immunity; simulation of latency	N/A	N/A	Not applicable.
3	MSM-specific disease burden	N/A	N/A	Not applicable.
3	Interaction between HIV and HPV	N/A	N/A	Not applicable.
3	Prior exposure	N/A	N/A	Not applicable.
4	HPV prevalence, CIN prevalence and cervical cancer incidence by HIV status	Y	F	Reported in a previous publication (Hall et. al.PLoS ONE 2020).[1]
4	HPV disease multipliers on HPV acquisition, progression from HPV infection to cancer (or relevant precursors, if modelled) for HIV-infected women/men	N/A	F	Reported in a previous publication (Hall et. al.PLoS ONE 2020).[1]
4	HPV-associated cancer mortality by HIV status (and CD4 count if modelled)	Y	F	Reported in a previous publication (Hall et. al.PLoS ONE 2020).[1]
4	Relevant co-morbidities	N/A	N/A	Not applicable.
4	HPV-associated screening sensitivity/specificity by HIV status	N/A	F	Reported in the appendix to the companion article ‘Benefits, harms and cost-effectiveness of cervical screening and treatment in 78 low-income and lower-middle income countries for women in the general population: modelling to support updated WHO cervical screening and

				treatment guidelines to prevent cervical cancer' by Simms & Keane.
5	HIV prevalence rates, if endemic in country	Y	Both	Reported in a previous publication (Hall et. al.PLoS ONE 2020).[1]
5	Description of any opportunistic or pilot/demonstration screening projects ongoing	Y	F	Reported in the discussion.
6	Routine screening behaviour (routine and follow-<br>up and test-of-cure)	Y	F	Reported in methods sub-section titled 'Scenarios for evaluation' and 'Screening attendance and treatment delivery'.
6	Screening test(s) and colposcopy accuracies	N/A	F	Reported in the appendix to the companion article 'Benefits, harms and cost-effectiveness of cervical screening and treatment in 78 low-income and lower-middle income countries for women in the general population: modelling to support updated WHO cervical screening and treatment guidelines to prevent cervical cancer' by Simms & Keane.
6	Abnormal test management (primary and triage)	N/A	F	Reported in the appendix to the companion article 'Benefits, harms and cost-effectiveness of cervical screening and treatment in 78 low-income and lower-middle income countries for women in the general population: modelling to support updated WHO cervical screening and treatment guidelines to prevent cervical cancer' by Simms & Keane.
6	Diagnostic follow-up of abnormal tests	N/A	F	Reported in the appendix to the companion article 'Benefits, harms and cost-effectiveness of cervical screening and treatment in 78 low-income and lower-

				middle income countries for women in the general population: modelling to support updated WHO cervical screening and treatment guidelines to prevent cervical cancer' by Simms & Keane.
6	Management by disease grade (confirmed disease)	N/A	F	Reported in the appendix to the companion article 'Benefits, harms and cost-effectiveness of cervical screening and treatment in 78 low-income and lower-middle income countries for women in the general population: modelling to support updated WHO cervical screening and treatment guidelines to prevent cervical cancer' by Simms & Keane.
6	Sources of information for screening structure and parameterisation	N/A	F	Reported in the appendix to the companion article 'Benefits, harms and cost-effectiveness of cervical screening and treatment in 78 low-income and lower-middle income countries for women in the general population: modelling to support updated WHO cervical screening and treatment guidelines to prevent cervical cancer' by Simms & Keane.
6	Herd effect	N/A	F	Model is transmission dynamic, so inclusion of herd effects is intrinsic to the model structure.
6	Association between vaccination and screening uptake	N/A	N/A	Not applicable.
6	Fixed – variable costs	N/A	N/A	Not applicable.
7	Timing between doses (for 2-dose)	N/A	N/A	Not applicable.
	**Outputs**	**Reported by age? (Y/N)**	**Report by sex (F/M/Both)?**	**Comments**

CRS	Cancer incidence, mortality, life years, QALYs/DALYs (as appropriate)	Reported but not by age	F	Reported in the results sub-sections ‘Benefits’ and ‘Harm’.
CRS	HPV prevalence, pre-intervention	N/A	N/A	Outcome not reported.
CRS	CIN2/3 detected	Reported but not by age	F	Reported in the results sub-section titled ‘Harm’.
CRS	Sensitivity analysis on key inputs	Reported but not by age	F	Reported in results subsection 'Sensitivity analysis findings'.
CRS	Incremental cost-effectiveness ratios and costs saved	N/A	N/A	Outcome not reported.
1	Absolute reductions in HPV infections, and/or warts, post-vaccination	N/A	N/A	Outcome not reported.
1	Absolute reductions in CIN2+ post-vaccination	N/A	N/A	Outcome not reported.
1	Absolute reductions in invasive cancer (cervical and other HPV cancers, as relevant) post-vaccination	N/A	N/A	Outcome not reported.
4	Reduction in cervical cancer incidence over time by HIV status (and CD4 count and ART status if modelled)	Y	F	All outcomes reported are in HIV positive women.
7	Threshold cost per vaccine dose	N/A	N/A	Not applicable.
